# 4-Hydroxy-1α,25-Dihydroxyvitamin D_3_: Synthesis and Structure–Function Study

**DOI:** 10.3390/biom14050551

**Published:** 2024-05-03

**Authors:** Carole Peluso-Iltis, Noé Pierrat, Daniela Rovito, Judit Osz, Daisuke Sawada, Atsushi Kittaka, Gilles Laverny, Natacha Rochel

**Affiliations:** 1 Institute of Genetics and Molecular and Cellular Biology (IGBMC), 67400 Illkirch, France; caro@igbmc.fr (C.P.-I.); laverny@igbmc.fr (G.L.); 2 CNRS UMR 7104, 67400 Illkirch, France; 3 Inserm U1258, 67400 Illkirch, France; 4 University of Strasbourg, 67400 Illkirch, France; 5 Graduate School of Medicine, Dentistry and Pharmaceutical Sciences, Okayama University, 1-1-1 Tsushimanaka, Kita-ku, Okayama 700-8530, Japan; dsawada@okayama-u.ac.jp; 6 Faculty of Pharmaceutical Sciences, Teikyo University, Tokyo 173-8605, Japan; akittaka@pharm.teikyo-u.ac.jp

**Keywords:** vitamin D metabolites, synthesis, calcemia, structure–function

## Abstract

The active vitamin D metabolites, 25-hydroxyvitamin D_3_ (25D_3_) and 1,25-dihydroxyvitamin D_3_ (1,25D_3_), are produced by successive hydroxylation steps and play key roles in several cellular processes. However, alternative metabolic pathways exist, and among them, the 4-hydroxylation of 25D_3_ is a major one. This study aims to investigate the structure–activity relationships of 4-hydroxy derivatives of 1,25D_3_. Structural analysis indicates that 1,4α,25(OH)_3_D_3_ and 1,4β,25(OH)_3_D_3_ maintain the anchoring hydrogen bonds of 1,25D_3_ and form additional interactions, stabilizing the active conformation of VDR. In addition, 1,4α,25D_3_ and 1,4β,25D_3_ are as potent as 1,25D_3_ in regulating the expression of VDR target genes in rat intestinal epithelial cells and in the mouse kidney. Moreover, these two 4-hydroxy derivatives promote hypercalcemia in mice at a dose similar to that of the parent compound.

## 1. Introduction

**Figure 1:**
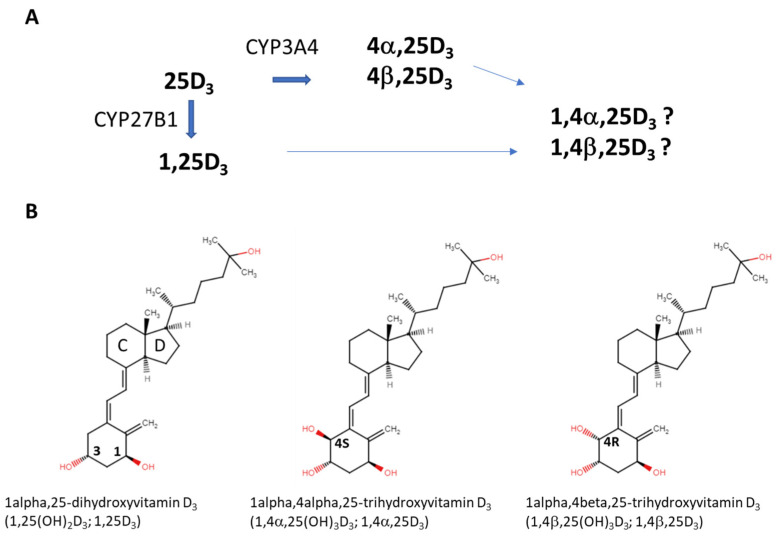
(**A**) Pathway of the production of 4-hydroxy metabolites of D_3_. (**B**) Chemical structures of the ligands.

The biologically hormonal form of vitamin D, 1α,25-dihydroxyvitamin D_3_ (1,25D_3_), regulates numerous biological processes, comprising calcium metabolism, cell growth, differentiation, anti-proliferation, apoptosis, and adaptive/innate immune responses [1,2,3]. These effects are mediated via the binding of the hormone to its nuclear receptor, VDR (NR1I1) [4]. The classical pathways of 1,25D_3_ activation involve 25-hydroxylation by a 25-hydroxylase and 1α-hydroxylation by CYP27B1 [5]. The general pathway associated with the degradation of 1,25D_3_ involves side chain oxidation by CYP24A1, leading to catabolic excreted vitamin D metabolites, including calcitroic acid, 1,25(OH)_2_D_3_-26,23S-lactone, and (23S,25R),25D_3_-(26,23)-lactone [6]. However, other metabolic and catabolic pathways are now recognized as being crucially important to vitamin D function and some metabolites have been shown to be biologically active [7,8]. Among them, 1α,3epi,25D_3_, a metabolite modified at the A-ring, has been shown to retain significant biological activity compared to 1,25D_3_ [9]. In addition, natural A-ring metabolites modified at C4 have also been reported. 4,25D_2_ was initially identified in the serum of rats intoxicated with high concentrations of vitamin D_2_ [10]. Further in vitro and in vivo studies have characterized the occurrence of a C4 hydroxylation pathway that involves the CYP3A4 enzyme [11,12]. Indeed, 4α,25D_3_ and 4β,25D_3_ are CYP3A4-catalyzed metabolites of 25D_3_ (Figure 1A). Whereas 4α,25D_3_ could only be detected in some studies, the levels of 4β,25D_3_, identified as an endogenous circulating metabolite in human plasma, are similar to those of 1,25D_3_ [11]. In addition, a significant accumulation of 4β,25D_3_ was observed in the plasma of healthy subjects treated with rifampin, a Pregnane X Receptor (PXR) agonist that induces CYP3A4 [12]. Moreover, high levels of 4β,25D_3_ were detected in patients exhibiting rickets and associated with a CYP3A4 mutation [13]. However, 1,25D_3_ is also a substrate for CYP3A4, leading to 1,23,25D_3_ inactivation [11,14]. 

Thus, 4-hydroxylation is a major pathway of 25D_3_ metabolism. However, the occurrence of the 4-hydroxylation of 1,25D_3_ by CYP3A4 remains to be demonstrated and the biological significance of 4-hydroxymetabolites remains elusive. The only available biological data concern synthetic 1,4α,25D_3_ and 1,4β,25D_3_ ligands (Figure 1B) and the results of luciferase reporter assays in transfected human osteosarcoma cells indicate that both isomers are less active than 1,25D_3_ [15,16]. Interestingly, significant differences in CYP24A1-induced 1,4,25D_3_ metabolism have been observed between the two isomers, and only the 4α isomer has been shown to be glucuronidated by certain hepatic UGT(s) [16,17]. In addition, 4β,25D_3_ has been shown to have greater metabolic stability and resistance to CYP24A1 than 4α,25D_3_ [18].

To further unveil the structure–activity relationships of 4-hydroxylated-1,25D_3_-VDR complexes, we describe a detailed synthetic route to the synthesis of 1,4α,25D_3_ and 1,4β,25D_3_ as well as some of the biological properties and crystal structures of their complexes with the VDR ligand binding domain (LBD).

## 2. Materials and Methods

### 2.1. Synthesis: General Information

All experiments were conducted under an argon atmosphere unless otherwise mentioned. All solvents and reagents were purified when necessary using standard procedure. Column chromatography was performed on silica gel 60 N (Kanto Chemical Co., Inc., Tokyo, Japan, 100–210 μm), flush column chromatography was performed on silica gel 60 (Merck, Tokyo, Japan, 0.040–0.063 mm), and preparative thin-layer chromatography was performed on silica gel 60 F_254_ (Merck, Tokyo, Japan, 0.5 mm). NMR spectra were measured on JEOL AL-400 (^1^H at 400 MHz) and ECP-600 (^13^C at 150 MHz) nuclear magnetic resonance spectrometers. Specific optical rotations were measured on a JASCO DIP-370 digital polarimeter.

### 2.2. Biochemistry

cDNA encoding His-tagged zVDR LBD (156–453) was cloned into pET28b. Recombinant proteins were produced in Escherichia coli BL21 DE3 and grown 3 h at 20 °C after induction with 1 mM IPTG at an OD600 of ~0.9. Soluble proteins were purified on Ni Hitrap FFcrude column (Cytiva, Marlborough, MA, USA), followed by His tag removal by thrombin cleavage and by size exclusion chromatography on HiLoad Superdex 75 column (Cytiva) equilibrated in Tris 20 mM pH7, NaCl 200 mM, and TCEP 1 mM. The proteins were concentrated to 3–7 mg/mL with an Amicon Ultra 10 kDa MWCO. 

### 2.3. Thermal Unfolding and Differential Scanning Fluorimetry (nanoDSF)

Fluorescence-based thermal experiments were performed using Prometheus NT.48 (NanoTemper Technologies, Munich, Germany) with capillaries containing 10 μL zVDR LBD at 3.2 mg/mL in the absence and presence of 2 equivalent ligands and/or 4 equivalents of the NCOA1 NR2 peptide (RHKILHRLLQEGSPS). The temperature was increased from 20 to 95 °C at a rate of 1 °C/min and fluorescence was measured at emission wavelengths of 330 nm and 350 nm. NanoTemper PR.Stability Analysis v1.0.2 was used to fit the data and to determine melting temperatures Tm. Triplicates of each sample were made. 

### 2.4. Fluorescence Polarization

Steady-state fluorescence polarization measurements were performed with a PHERAstar Plus (BMG Labtech, Champigny s/Marne, France) spectrofluorometer. Titrations were carried out by adding increasing zVDR LBD concentrations to 10 nM Fluorescein-NCOA1 NR2 peptide (FL-LTARHKILHRLLQEGSPSD) in 50 mM Hepes pH8.0, 50 mM NaCl, 2% glycerol, 1 mM TCEP, and 0.05% Tween-20. The binding affinity of the FL-peptide to zVDR LBD was quantified in response to 1,25D_3_, 1,4α,25D_3_, or 1,4β,25D_3_. The excitation wavelength was 488 nm and the emitted light was monitored through high-pass filters (520 nm). The dissociation constant (Kd) was calculated using GraphPad Prism 10 by fitting an agonist vs. response equation. All experiments were performed at 25 °C and the results are representative of three distinct experiments.

### 2.5. Crystallization and Structure Determination

The concentrated protein at 5 mg/mL was incubated with a 2-fold excess of ligand and a 3-fold excess of the coactivator NCOA2 (KHKILHRLLQDSS) peptide. Crystallization experiments were carried out via sitting drop vapor diffusion at 290 K by mixing equal volumes (0.2µL) of the protein–ligand complexes and of the reservoir solution (0.1 M MES pH 6.0,2.5 M NaOAc). The crystals of the complexes were transferred to an artificial mother liquor containing 0.1 M MES at pH 6.0 and 3 M NaOAc and were flash-cooled in liquid nitrogen. Data on the crystals of zVDR-1,4α,25D_3_ and of zVDR-1,4β,25D_3_ were collected on Proxima2 beamline at Soleil synchrotron. Crystallographic raw data were processed with XDS [19] and scaled with AIMLESS [20]. The structures were solved and refined using Phenix [21] and iterative model building using COOT [22]. Crystallographic refinement statistics are presented in Supplementary Table S1.

### 2.6. Cells

IEC-18 rat intestinal epithelial cells (American Type Culture Collection, Rockville, MD, USA, CRL-1589, RRID:CVCL_0342) were grown in Dulbecco’s modified Eagle’s medium (DMEM) 4.5 g/L glucose supplemented with 5% fetal calf serum (FCS), 1 mM sodium pyruvate, 0.1 UI/mL insulin, and 40 µg/mL gentamicin. Cells at 80% confluency were grown in a medium supplemented with 5% charcoal-treated FCS for 24 h and treated as indicated.

### 2.7. Mice

A cohort of C57BL6J mice aged between 8 and 12 weeks were treated per os with 1 μg/kg/day of 1,25D_3_, 1,4α,25D_3_, or 1,4β,25D_3_ in 100 μL of oil daily for 4 days [23]. On day 4, blood and kidneys were harvested for analysis. A cohort of mice treated with 100 μL of oil were used as a control. All animal experimental protocols were conducted in compliance with French and EU regulations on the use of laboratory animals for research and approved by the IGBMC Ethical Committee and the French Ministry for National Education, Higher Education, and Research (#10047-2017052615101492).

### 2.8. Functional Assay

Total RNA from IEC-18 cells and kidneys were isolated using TRI Reagent (Molecular Research Center, Inc., Euromedex, France) according to the manufacturer’s protocol. RNA was quantified by spectrophotometry (Nanodrop, Thermo Fisher, Illkirch, France) and cDNA was prepared using 2 µg of total RNA, random hexamers, and SuperScript IV reverse transcriptase (Thermo Fisher, Illkirch, France) following the manufacturer’s instructions. Quantitative PCR (Light Cycler 480-II) was performed using the Light Cycler 480 SYBR Green I Master X2 Kit (Roche Diagnostics, Meylan, France) according to the supplier’s protocol. Data were analyzed using the standard curve method following the manufacturer’s protocol (Lightcycler 480 II, Roche Diagnostics, Meylan, France), and the *18S* housekeeping gene was used as an internal control. The sets of primers used were as follows: *Cyp24a1* (rat), 5′-TCCATGAGGCTTACCCCAAG-3′ (sense), and 5′-GCGTATTCACCCAGAACCGT-3′ (antisense); *S100g* (rat), 5′-GACAGCAAGCAGCACAGAAAA-3′ (sense), and 5′-TGGACAGCTGGTTTGGATCG-3′ (antisense); *Trpv6* (rat), 5′-GAGCACAGGTTGTGGCTACT-3′ (sense) and 5′-CCAAGACCATACTCTCGCCC-3′ (antisense); *18S* (rat), 5′-AGCTCACTGGCATGGCCTTC-3′ (sense), and 5′-CGCCTGCTTCACCACCTTC-3′ (antisense); *Cyp24a1* (mouse), 5′-GGCGGAAGATGTGAGGAATA-3′ (sense), and 5′-GCCCAGCACTTGGGTATTTA-3′ (antisense); *Cyp27b1* (mouse), 5′-CGTCCAGAGCGCTGTAGTT-3′ (sense), and 5′-TCTTCACCATCCGCCGTTAG-3′ (antisense); *Trpv5* (mouse), 5′-TGGTGGGTCAGAGACCAAG-3′ (sense), and 5′-CAGTGGAGACTCCCAAATACTTTT-3′ (antisense); *18s* (mouse), 5′-AGCTCACTGGCATGGCCTTC-3′ (sense), and 5′-CGCCTGCTTCACCACCTTC-3′ (antisense).

### 2.9. Serum Calcium Levels

Mouse blood was collected in Microvette^®^ 500 lithium heparin (SARSTEDT) and centrifuged at 400 g for 10 min at 4 °C. The supernatant corresponding to the serum was retained. Serum calcium levels were determined using a colorimetric assay (MAK022, Sigma Aldrich, St. Quentin Fallavier, France) in accordance with the supplier’s instructions. 

## 3. Results and Discussion

### 3.1. Synthesis

**Scheme 1:**
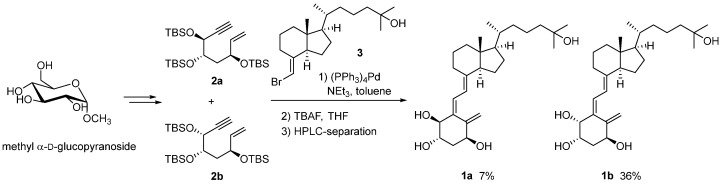
Synthesis of 1α,4α,25-trihydroxyvitamin D_3_ (**1a**) and 1α,4β,25-trihydroxyvitamin D_3_ (**1b**).

Compounds 1,4α,25D_3_ (**1a**) and 1,4β,25D_3_ (**1b**) were synthesized as follows: the A-ring precursors enyne **2a** and **2b** were synthesized from methyl α-D-glucopyranoside by our reported procedures [17], and stereochemistry at the C4-position (4*R*) of major product **2b** was determined by the modified Mosher’s method (Scheme 1) [16,24]. 

Triethylamine (1.9 mL) and Pd(PPh_3_)_4_ (21 mg, 190 μmol) were added to a solution of the enyne mixture (without the separation of **2a** and **2b**, 95 mg, 190 μmol, **2a**/**2b** = 1/3) and bromoolefin **3** (81 mg, 230 μmol) in toluene (1.9 mL) at room temperature. The reaction mixture was stirred at 80 °C overnight. After cooling to room temperature, the mixture was concentrated in vacuo. The residue was purified by flush column chromatography on silica gel (hexane/EtOAc = 20/1) to give the coupling products of silyl-protected **1a** and **1b** as an inseparable mixture (63 mg, 43% from enynes) as a pale yellow oil. This was used for the next reaction without further purification.

TBAF (0.4 mL, 1.0 M solution in THF, 400 μmol) was added to a solution of the mixture of silyl-protected **1a** and **1b** (30 mg, 39 μmol) in THF (0.8 mL) at 0 °C. The reaction mixture was stirred at room temperature for 3 h, and then at 80 °C for 5 h. After cooling, the reaction was quenched with water at 0 °C. The mixture was extracted with EtOAc, and the organic layer was washed with brine, dried over Na_2_SO_4_, filtered, and concentrated. The residue was purified by preparative silica gel TLC plate (hexane/EtOAc = 2/1) to give the mixture of **1a** and **1b** (17 mg, quant) as a pale yellow oil. The mixture was re-purified and separated by reversed-phase HPLC (YMC-Pack ODS column, 20 × 250 mm, CH_3_OH/H_2_O = 9/1) to give **1a** (1.2 mg,) and **1b** (6.4 mg), each as a colorless oil [16].

### 3.2. Biological Assays

**Figure 2:**
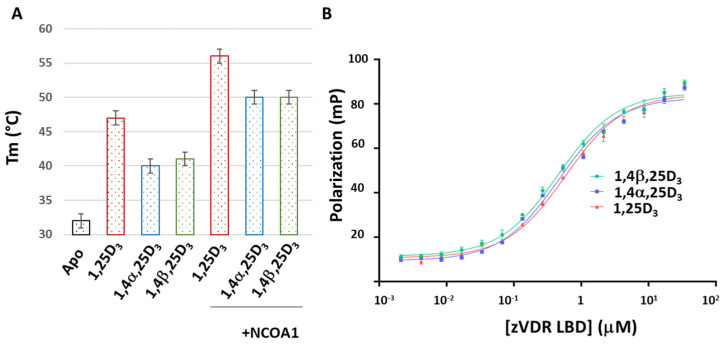
Effect of ligands on VDR stability and interaction with NCOA1. (**A**) Thermal stability of purified zVDR LBD alone and upon binding to ligands in absence or presence of NCOA1 NR2 coactivator peptide. (**B**) 4-hydroxylated-1,25D_3_-bound VDR LBD efficiently recruits NCOA1 coactivator peptide. Titration of fluorescein-NCOA1 peptide with zVDR LBD in complex with 1,25D_3_ (in red), 1,4α,25D_3_ (in blue), or 1,4β,25D_3_ (in green).

Nano differential scanning fluorimetry analysis confirmed the binding of the 1,4α,25(OH)_3_D_3_ and 1,4β,25(OH)_3_D_3_ ligands to the zebrafish (z)VDR LBD, which has been shown to bind ligands similarly to the human VDR [25]. The two 4-hydroxylated ligands increased the stabilization of the zVDR LBD, which was further increased in the presence of a NCOA1 coactivator peptide, which encompasses one nuclear receptor LXXLL interacting motif (Figure 2A). However, the stabilization of the complexes was weaker compared to the effect of 1,25D_3_ in this assay. The interaction of the LBD of zVDR with the NCOA1 coactivator peptide was next quantified by fluorescence polarization in the presence of saturating ligand concentrations of 1,25D3, 1,4α,25D_3_, or 1,4β,25D_3_ (Figure 2B). Whereas the zVDR LBD in its apo form did not bind the NCOA1 peptide [26], the NCOA1 peptide bound to zVDR LBD with a similar affinity in the presence of the tested ligands (Kd = 0.54 µM, 0.50 µM, and 0.48 µM for the 1,25D_3_, 1,4α,25D_3_, and 1,4β,25D_3_ complexes, respectively). 

**Figure 3:**
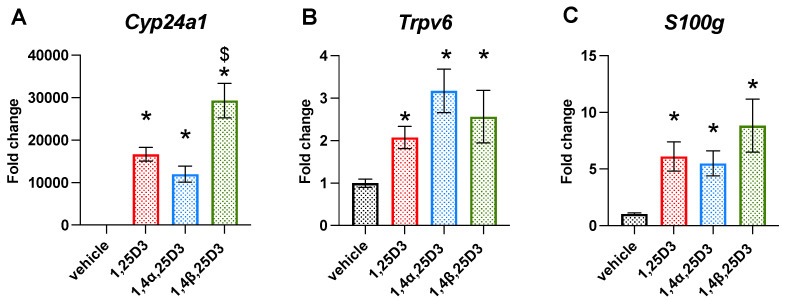
Relative transcript levels of the VDR target genes *Cyp24a1* (**A**), *Trpv6* (**B**) and *S100g* (**C**) in IEC-18 cells treated for 24 h with vehicle or 100 nM of the indicated ligands, determined by RT-qPCR (n = 4 biological replicates). *: *p* < 0.05 vs. Veh and $: *p* < 0.05 vs. 1,25D_3_. One-way ANOVA followed by Tukey’s test.

The effects of these 4-hydroxylated ligands on VDR activities were previously studied using reporter gene assays [15,16]. To determine their potency to induce endogenous VDR target genes in intestinal cells, a key vitamin D target tissue, we evaluated the expression of the transcript levels of several VDR target genes (*Cyp24a1*, *Trpv6*, and *S100g*) in rat intestinal epithelial (IEC-18) cells treated for 24 h with vehicle or 100 nM of 1,25D_3_, 1,4α,25D_3_, and 1,4β,25D_3_ by RT-qPCR. The transcript levels of these VDR target genes were induced at least twofold in the presence of the tested compounds compared to vehicle-treated cells (Figure 3). Consequently, 4-hydroxylated compounds are potent VDR agonist ligands with a potency comparable to that of 1,25D_3_. Note that *Cyp24a1* transcripts were higher in 1,4β,25D_3_-treated cells than in 1,25D_3_- and 1,4α,25D_3_-treated cells, indicating that 1,4β,25D_3_ is more potent. Thus, in contrast to the previous study using reporter assays in VDR-transfected cells, the two metabolites enhanced endogenous VDR activities in IEC-18 cells.

**Figure 4:**
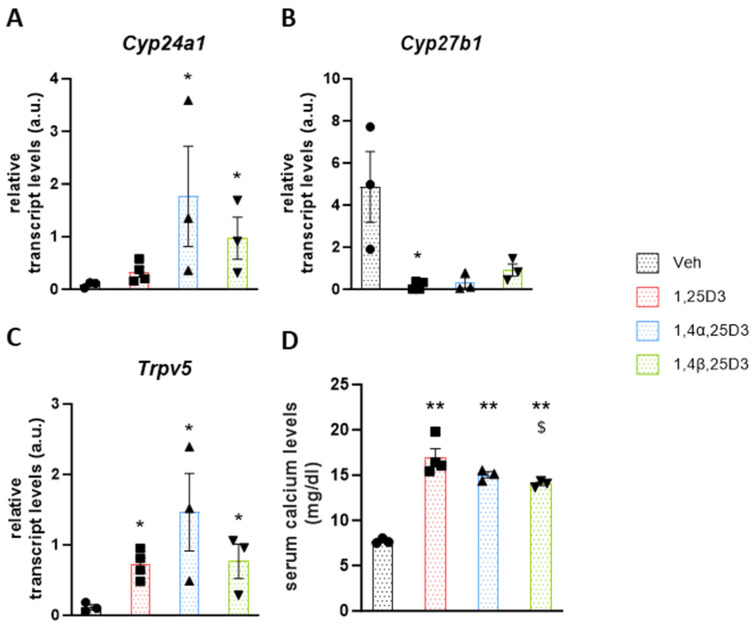
Relative renal transcript levels of the VDR target genes *Cyp24a1* (**A**), *Cyp27b1* (**B**), and *Trpv5* (**C**) and serum calcium levels (**D**) in WT mice treated with vehicle (Veh) or 1 µg/kg/day of the indicated ligands daily for 4 days determined by RT-qPCR and colorimetric test, respectively (n = 3–4; each point represents an animal). **: *p* < 0.001 and *: *p* < 0.05 vs. Veh; $: *p* < 0.05 vs. 1,25D_3_. One-way ANOVA followed by Tukey’s test.

The results in IEC18 cells prompted us to investigate the in vivo activities of 1,4α,25D_3_ and 1,4β,25D_3_. We determined the effects of 1,4α,25D_3_ and 1,4β,25D_3_ administration on the expression of the VDR target genes *Cyp24a1* and *Cyp27b1*, two genes encoding proteins involved in the metabolic pathway of vitamin D and known to be upregulated and downregulated by 1,25D_3_, respectively. After treatment with the 4-hydroxylated metabolites, Cyp24a1 transcript levels were induced, whereas the expression of Cyp27b1 decreased (Figure 4A,B). The expression of Trpv5, a VDR target gene encoding for a channel involved in kidney calcium reabsorption, was induced by 1,4α,25D_3_ or 1,4β,25D_3_ with a similar potency to that of 1,25D_3_ (Figure 4C). Then, we determined the pro-calcemic activities of the 4-hydroxylated metabolites. In accordance with previous results, mice treated with 1 µg/kg/day of 1,25D_3_ for 4 days were hypercalcemic [23]. Interestingly, serum calcium levels after 1,4α,25D_3_ or 1,4β,25D_3_ intoxication were similar to those in 1,25D_3_-treated mice. All together, these results indicate that 1,4α,25D_3_ or 1,4β,25D_3_ have comparable in vivo activities to those of 1,25D_3_ (Figure 4D).

### 3.3. Ligand Binding Mode to VDR

**Figure 5:**
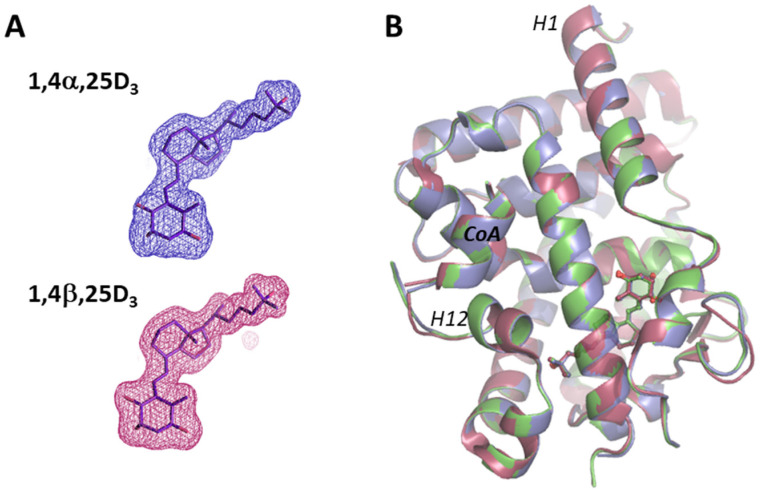
Crystal structures of the zVDR complexes with 1α,4a,25(OH)_3_D_3_ and 1a,4b,25(OH)_3_D_3_. (**A**) Ligands modeled into omit Polder maps contoured at 3σ. (**B**) Superimposed protein structures of 1,4a,25D_3_ (blue) and 1,4b,25D_3_ (green) compared to the 1α,25D3 zVDR complex (red).

To decipher the ligand binding mode of the two 4-hydroxy metabolites to VDR, we solved the crystal structures of their complexes with the zVDR LBD in the presence of a NCOA2 coactivator peptide. The structures of the zVDR LBD bound to 1,4α,25D_3_ and to 1,4β,25D_3_ were determined at a resolution of 1.95 and 1.8 Å, respectively. The crystallographic data are summarized in Supplementary Table S1. After the refinement of the protein alone, the map showed an unambiguous electron density in which the ligands fit (Figure 5A). The complexes formed by the zVDR LBD bound to the two 4-OH compounds adopt the canonical active conformation, as described in all previously reported agonist-bound VDRs (Figure 5B). The conformation of the activation helix 12 is strictly maintained and the coactivator peptide forms similar interactions as in the complex with 1,25D_3_. When compared to the structure of the zVDR LBD-1,25D_3_ complex, the atomic coordinates of zVDR LBD bound to 1,4α,25D_3_ and 1,4β,25D_3_ show a very small root mean square deviation of 0.3 Å over 235 Cα atoms, reflecting their high structural homology.

**Figure 6:**
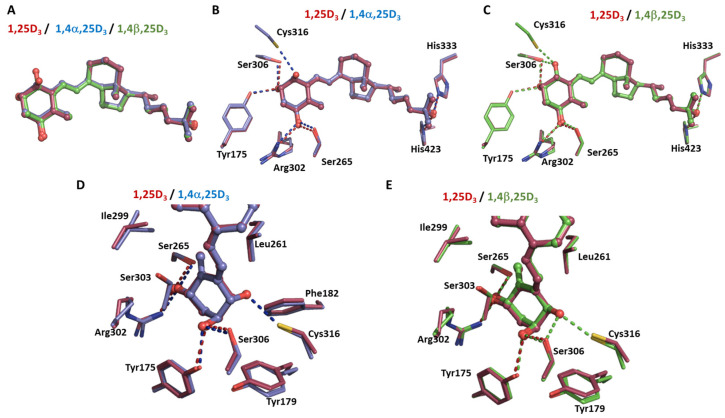
Ligand binding mode. (**A**) Conformation of the bound ligands in the VDR LBD complexes. Superimposed ligands, 1,25D_3_ (red), 1,4α,25D_3_ (blue), and 1,4β,25D_3_ (green) are shown in stick representation. (**B**) Hydrogen bonds formed by the hydroxyl groups of 1,4α,25D_3_ (blue) and 1,25D_3_ (red) in the LBP of the zVDR. (**C**) Hydrogen bonds formed by the hydroxyl groups of 1,4β,25D_3_ (green) and 1,25D_3_ (red) in the LBP of the zVDR. (**D**,**E**) Close-up view of the superimposed ligands around the A-ring of the ligands: 1,4α,25D_3_ (blue), 1,4β,25D_3_ (green), 1,25D_3_ (red). Residue side-chains closer than 4.0 Å contacting the ligands are shown. The hydrogen bonds formed by the ligands are shown as dashed lines.

The addition of an additional hydroxyl group on C4 does not modify the A-ring conformation of the ligand, and the seco-B and C/D-rings and aliphatic side chain have similar conformations to those of 1,25D_3_ (Figure 6A). The distances between C1-OH and C25-OH are 13.12Å, 13.11Å, and 12.95Å, and between the C3-OH and C25-OH they are 15.36 Å, 15.38 Å, and 15.28 Å for 1,25D_3_, 1,4α,25D_3_, and 1,4β,25D_3_, respectively. The interactions with the seco-B, C/D-rings and the side chain of the two analogs are similar to those formed by 1,25D_3_ as well as the hydrogen bonds formed with 1-OH, 3-OH, and 25-OH (Figure 6B,C). Differences are observed around the 4-OH group; the 4-OH group of the 4β hydroxylated compound forms hydrogen bonds with Ser306 and Cys316, whereas its diastereomer 4α acts through hydrogen bonding only with Cys316 but forms stronger van der Waals interactions with Phe182 and Leu261 (Figure 6D,E). To adapt the additional hydroxyl group, some side chain amino acid residues, notably Tyr179, Phe182, and Cys316, are shifted slightly, by 0.4–0.6 Å. These structural data agree well with the induced biological activity of the two compounds, which are similar to the natural hormone.

## 4. Discussion

In the present work, we show that 4-hydroxylated 1,25D_3_ ligands are potent VDR agonists.

The most active form of vitamin D_3_ is 1,25D_3_, produced by two sequential hydroxylations of vitamin D_3_, which can be obtained from dietary sources or synthesized endogenously in the skin after the photolytic conversion of 7-dehydrocholesterol [4,5]. But 25D_3_ is not only a metabolic precursor of 1,25D_3_: it also acts as a VDR agonist itself, with gene regulatory and anti-proliferative properties [27,28,29]. Indeed, 25D_3_, when present at high concentrations, induces VDR activities, notably in cancer cells when 1,25D_3_ production is abolished [28,29,30]. However, 25D_3_ binds to VDR less efficiently than 1,25D_3_ [31], indicating that the hydroxyl group at C1 has a critical role in achieving high affinity. 

The recent identification of alternative vitamin D_3_ pathways and of the major enzymes involved has revealed the existence of other natural vitamin D_3_ metabolites, notably with modifications of the A-ring [7,8]. The 3-epimer of 1,25D_3_ has been shown to retain significant biological activity compared to the natural hormone, although its activity is lower than that of 1,25D_3_ [7,32]. The crystal structural analysis of VDR-1,25-3-epiD_3_ showed that 1,25-3-epi-D_3_ takes a slightly more compact conformation in the VDR ligand binding pocket and compensates for the loss of interaction of the 3-OH group with hSer278 by a water-mediated hydrogen bond [32]. Here, we show that the 4-hydroxylated 1,25D_3_ compounds maintain all anchoring H-bonds of 1,25D_3_ and form additional interactions_._ The 4-OH group of the 4β-hydroxylated compound forms hydrogen bonds with zSer306 and zCys316, whereas its diastereomer 4α acts through hydrogen bonding only with zCys316, but forms stronger van der Waals interactions with zPhe182 and zLeu261. These interactions explain why those compounds are as active as 1,25D_3_. In IEC-18 cells, the two compounds are potent VDR agonist ligands, with similar activities to 1,25D_3_. In addition, they regulate the expression of renal VDR target genes and increase serum calcium levels, demonstrating that these 4-hydroxylated metabolites enhance VDR activities in vivo. While 4,25D_3_ are endogenous metabolites catalyzed by CYP3A4 [11,12], the occurrence of 1,25D_3_ C4-hydroxylation or 4,25D_3_ C1-hydroxylation remains to be demonstrated. The enzymatic activity of CYP3A4 was associated with 1,25D_3_ inactivation via C-23 hydroxylation [11,14]. However, other presumed hydroxylated “degradation” products have demonstrated transcriptional activity [33].

Their high and differential metabolic stability compared to that of 1,25D_3_, together with their significant biologic activity, makes these synthetic 4-hydroxylated 1,25D_3_ analogs promising candidate ligands for clinical applications. However, their target specificity needs further investigation in future studies. In addition, this study provides information for developing novel VDR agonists. Thousands of 1,25D_3_ analogs have already been synthetized, and some modifications, such as C20 epimer, C-2 substitutions, or side chain rigidification, have been shown to improve the stability of VDR complexes and ligand-induced activities [34]. The incorporation of a 4-hydroxyl group into secosteroidal ligands could provide new potent VDR agonists. 

## 5. Conclusions

In this study, two 4-hydroxylated analogs of 1,25D_3_ were chemically synthetized. 1α,4α,25D_3_ and 1α,4β,25D_3_ ligands showed VDR gene regulatory activities similar to 1,25D_3_. The crystal structures of zVDR LBD in complex with the two epimers provide a mechanistic insight for the specific recognition of 4-hydroxylated metabolites of 1,25D_3_. Therefore, we conclude that the C4-hydroxylation pathway produces active metabolites with similar biochemical and biological properties to those of 1,25D_3_.

## Data Availability

Coordinates and structure factors are deposited into the Protein Data Bank with accession numbers 9EZ1 for zVDR-1,4α,25D_3_ and 9EZ2 for zVDR-1,4β,25D_3_.

## Supplementary Materials

The following supporting information can be downloaded at: https://www.mdpi.com/article/10.3390/biom14050551/s1, Table S1: Crystallographic data and refinement.
