# Supplementary material for: 4-Hydroxy-1α,25-Dihydroxyvitamin D3: Synthesis and Structure–Function Study
Source: Biomolecules. 2024 May 3;14(5):551. doi: 10.3390/biom14050551 (PMC11117473; doi:10.3390/biom14050551)
Supplement: Supplementary file 1 [file biomolecules-14-00551-s001.zip › biomolecules-2979293-supplementary.pdf]

	zVDR LBD – 1,4α,25D$_3$	zVDR LBD – 1,4β,25D$_3$
**PDB ID**	9EZ1	9EZ2
**Data collection**		
Beamline	PX2	PX2
Space group	$P6_522$	$P6_522$
Unit-cell parameters (Å, °)	65.98, 65.98, 263.47<br>90, 90, 120	66.14, 66.14, 263.74<br>90, 90, 120
Resolution range (Å)	43.16-1.95	43.96-1.8
Unique reflections	25896	32871
I/σ(I)	22.96	23.45
$CC_{1/2}$	1	1
Completeness	99.30	99.30
**Refinement**		
Resolution range (Å)	43.16-1.95	43.96-1.8
R_{work} / R_{free} (%)	20.58 / 25.52	20.16 / 22.98
Ramachandran		
Allowed (%)	1.23	0.82
Favored (%)	98.77	99.18
Mean B factors (Å^2)		
Protein	61.31	58.52
Ligand	42.56	39.18

Supplementary Table S1. Crystallographic data and refinement.
